# Comparative Study on Volatile Compounds and Taste Components of Different Durian Cultivars Based on GC-MS, UHPLC, HPAEC-PAD, E-Tongue and E-Nose

**DOI:** 10.3390/molecules27041264

**Published:** 2022-02-14

**Authors:** Zuobing Xiao, Minxing Niu, Yunwei Niu

**Affiliations:** 1 Shanghai Institute of Technology, School of Perfume and Aroma Technology, Shanghai 201418, China; niuge211@sina.com (Z.X.); niuminxing@gmail.com (M.N.); 2 School of Agriculture and Biology, Shanghai Jiao Tong University, Shanghai 200240, China

**Keywords:** durian, SPME, HPAEC-PAD, UHPLC, e-nose, e-tongue, PCA

## Abstract

In order to comprehensively evaluate the aroma-active substances and taste components of durian, solid-phase microextraction combined with gas chromatography mass spectrometry (SPME/GC-MS), high-performance anion-exchange chromatography with pulsed amperometric detection (HPAEC-PAD) and ultra-high-performance liquid chromatography (UHPLC) were used to test the key components of three popular durian cultivars. A total of 27 volatile compounds, 5 sugars, 27 organic acids and 19 free amino acids were detected in Black Thorn (BT) durian. A total of 38 volatile compounds, 4 sugars, 27 organic acids and 19 free amino acids were detected in Monthong (MT) durian. A total of 36 volatile compounds, 4 sugars, 27 organic acids and 20 free amino acids were detected in Musang King (MK) durian. Finally, the flavor differences of the three durians were evaluated using electronic nose (e-nose) and electronic tongue (e-tongue), and different cultivars were classified through principal component analysis (PCA).

## 1. Introduction

Durian (*Durio zibethinus* Murr.) belongs to the Malvaceae family and is one of the most economically important fruits in Southeast Asia. Thailand and Malaysia are important producing areas of durian. MT is one of the well-known durian cultivars grown commercially in Thailand [1]. The output of durian in 2019 exceeded one million tons, and it is exported mainly to China and the world market [2]. Malaysia is also an important producer and exporter of durian and is second only to Thailand in terms of output and export value. MT and MK are well-known durian cultivars in Malaysia, with a high degree of commercial cultivation, and their unique smell and creamy flavor are deeply loved by consumers [3]. The prices of the three durians are significantly different (BT > MK > MT). To sum up, three different durians were selected for research based on price and popularity.

The quality of fruits is often determined by multiple factors, such as color, texture, aroma and taste. Most of the previous papers studied aroma compounds and taste components separately. In fact, however, taste and aroma influence each other. Oladokun et al. [4] found that the aroma of hops can significantly affect the taste of beer. The bitterness and astringency of beer with the aroma of Hersbrucker hop were significantly enhanced. Arvisenet et al. [5] found that sugar and acid in a certain concentration range can enhance the aroma of wine. The aroma components and taste components in durian could influence each other, creating the unique aroma and taste of durian. Li et al. [6,7] identified 44 aroma-active substances of MT durian through solvent-assisted flavor evaporation (SAFE) combined with GC-MS. Through stable isotope dilution analysis (SIDA) combined with threshold values, they found that ethyl 2-methylbutyrate and ethyl mercaptan had high odor activity values (OAV). Yi et al. [3] identified 23 volatile compounds in BT and 24 volatile compounds in MK through SPME combined with GC-MS. Three sugars and six organic acids were identified by high-performance liquid chromatography (HPLC) combined with mass spectrometry. Pinsorn et al. [1] used capillary electrophoresis time-of-flight mass spectrometry (CE-TOF/MS) to study the metabolites of durian pulp flavor, such as amino acids. They found that the metabolite profiles of durian from different cultivars were significantly different, which may be an important reason for the difference in durian flavor.

In this paper, SPME was used to extract the aroma-active substances in durian. A total of 27, 38 and 36 volatile compounds were detected in BT, MT and MK, respectively. SPME is widely used in fruit aroma extraction, because it is fast, simple and solvent-free. HPAEC-PAD was used to determine sugar content in durian pulp. This method optimizes the analysis process and shortens the analysis time compared with HPLC [8]. A total of five, four and four sugars were detected in BT, MT and MK, respectively. UHPLC was used to determine the organic acids and amino acids in durian pulp with higher sensitivity and shorter analysis time than traditional HPLC [9]. A total of 27 organic acids and 20 free amino acids were detected in the three durians (except for the lack of L-Tryptophan in BT).

The purpose of this study is to detect aroma-active components and taste components of three durian cultivars, qualitatively and quantitatively, combined with e-nose and e-tongue to evaluate their flavor differences. This work aims at providing a reference for establishing a rapid and comprehensive analysis for durian aroma components and taste components, and at applying it to durian-flavored food. Theoretical bases for the aroma and taste improvement of durian-flavored foods are provided in this paper.

## 2. Results and Discussion

### 2.1. Volatile Organic Compounds (VOCs)

In this study, 49 volatile compounds were detected in three durian cultivars, including 22 ester compounds, 13 sulfur compounds, 6 alcohol compounds, 3 ketone compounds, 3 aldehyde compounds and 2 olefin compounds. As shown in Table 1, a total of 27, 38 and 36 compounds were detected in BT, MT and MK, respectively. Methyl 2-methyl butyrate, ethyl 2-methyl butyrate, propyl 2-methyl butyrate, hexyl hexanoate, methyl octanoate and propyl tiglate existed in all durian cultivars. Propyl 2-methyl butyrate (1818.42 μg/kg) was the most abundant ester compound in BT durian, and it was also the characteristic ester of Chanee durian [10]. It has also been found in sea buckthorn [11] and apple [12], and has aromas of wine, apple and pineapple. The ester compound with the highest content in MT and MK was ethyl 2-methyl butyrate, whose contents were 11,680.31 μg/kg and 14,484.20 μg/kg, respectively. Ethyl 2-methyl butyrate has been described as a tropical fruit aroma and it is a key component of the aroma of durian [7]. Ethyl propionate, ethyl (*Z*)-2-crotonate and hexyl 2-methyl butyrate were only detected in BT. Ethyl acetate, diethyl carbonate, ethyl butyrate, hexyl formate and ethyl valerate were only detected in MT, which may be one of the reasons for the differences in the aroma of different durian cultivars.

Ethyl mercaptan, methyl ethyl disulfide, diethyl disulfide, methyl propyl disulfide, dipropyl disulfide, diethyl trisulfide, 3,5-dimethyl-1,2,4-trithiolane and acetaldehyde diethyl mercaptal were detected in all durian cultivars. Diethyl disulfide was the most abundant sulfur-containing compound in all three durian cultivars, and was 17,777.04 μg/kg, 8665.01 μg/kg and 27,253.26 μg/kg in BT, MT and MK, respectively. Diethyl disulfide is usually described as the smell of onion or garlic, and it was transformed from the less stable ethyl mercaptan and propyl mercaptan [13]. 3,5-dimethyl-1,2,4-trithiolane and acetaldehyde diethyl mercaptal are widespread in many durian cultivars. They are rare in other fruits, but they are important aroma compounds in durian. Methyl ethyl sulfide, S-ethyl thioacetate and dipropyl sulfide were only detected in MK, which may be one of the reasons for the differences in aroma of different durian cultivars.

Diacetyl and acetoin are important aroma compounds in durian as well as in cheese, butter, yogurt and butter [14], and have been described as the smell of milky and sweet flavor. It is worth mentioning that heptanal (71.22 μg/kg) and 1-octanal (81.84 μg/kg) were only detected in MT. Heptanal has a grassy aroma, and has also been detected in persimmon and melon [15,16]. Octanal has the aroma of citrus and orange, and is the main aroma compound in citrus [17]. The difference in aroma components between different durian cultivars may be caused by the differences in variety and growth environment. The light time temperatures during the day and at night and the rainfall during the growth stage may cause the differences in contents and types of the final aroma compounds of durian [18].

### 2.2. Sugars, Organic Acids and Amino Acids of Durian Fruits

Sweetness is considered a key factor that determines the quality of fruit, and the taste of the fruit is directly affected by the sugar content [19]. Sweetness is also an index of fruit maturity, and sucrose, fructose and glucose play an important role at this stage [20]. For example, during the ripening process of green bananas, amylase- and sucrose-synthesis-related enzymes degrade starch into sucrose [21]. Sucrose is a substance that produces a sense of sweetness. Table 2 shows the content difference of glucose, fructose, maltose, ribose and sucrose in different durian cultivars. The concentration of sucrose in each durian variety is significantly higher than that of other sugars. The sucrose content of BT is about 2–5 times higher than that of the other two cultivars. The fructose and glucose contents of MK and MT are similar, while the contents of fructose and glucose of BT are the lowest. Traces of ribose (0.69 μg/mg) were also detected in BT, which is not found in the other two cultivars. The differences in the contents of glucose and fructose in different cultivars are caused by differences in the activities of related enzymes [22].

The contents of organic acids vary greatly in different varieties of fruits. Some organic acids in fruit pulp act as important precursor substances in certain metabolic pathways. Organic acids are also important flavor substances, and their contents affect the palatability of the fruits [23]. A total of 27 organic acids were detected in three durians. Malic acid, citric acid, tartaric acid, succinic acid, acetic acid and lactic acid are common organic acids in durian [24]. Table 2 shows the differences in the content of organic acids in different durian cultivars. Succinic acid is the organic acid with the highest content in the three durian cultivars, and the content in BT is the highest (11,453.95 ng/mg). In addition, among the 27 organic acids, malic acid, acetic acid, citric acid, maleic acid and propionic acid are predominant. These organic acid contents are beyond 1000 ng/mg, except for the propionic acid in MT which is 275.93 ng/mg. Although the content of tartaric acid is low (72.44–221.67 ng/mg), it has a better comprehensive taste performance than other organic acids such as citric acid in the interaction of sweet and sour [25]. Butyric acid, valeric acid, hexanoic acid and octanoic acid in durian have been detected by gas chromatography combined with mass spectrometry in the literature [26]. Quinic acid is the main organic acid in citrus fruits, and can account for 20–30% of the dry weight of the pulp [27]. Studies have shown that the concentration and types of organic acids affects the flavor and palatability of fruits [28].

Amino acid and its derivatives are important contributors to fruit flavor [29]. Table 2 summarizes the contents of 20 common amino acids. The contents of aspartic acids in the flesh of BT and MK were 6708.92 ng/mg and 4631.31 ng/mg, respectively, which were the highest concentrations compared with other amino acids. The free amino acid with the highest content in MT is alanine (6019.09 ng/mg). Among the 20 amino acids, alanine, aspartic acid, glutamic acid and leucine are predominant. These amino acid contents are beyond 2000 ng/mg, except for the leucine in MT, which is 1903.56 ng/mg. Free tryptophan was not detected in BT and MT, and the content in MK is extremely low (0.15 ng/mg). In previous research [30,31], amino acids were classified as sour (aspartic acid), sweet (alanine, glycine, serine and threonine), bitter (arginine, leucine, isoleucine, methionine and valine) and umami (glutamic acid) according to their contribution to the taste of durian. The remaining nine amino acids showed extremely mild taste or were tasteless. The contents of sweet amino acids, sour amino acid, bitter amino acids and umami amino acid in the flesh of BT were found to be 11,005.99 ng/mg, 6708.92 ng/mg, 3849.50 ng/mg and 6447.98 ng/mg, respectively, through calculations. In BT, the amino acid contents of the four types are higher than those of the other two cultivars. Meanwhile, some amino acids are also precursors of key aroma substances in fruits. For example, the methionine γ-lyase in durian converts methionine into methyl mercaptan, which is the key to the formation of durian characteristic odor [32].

### 2.3. E-Nose and E-Tongue Analyses

**Figure 1:**
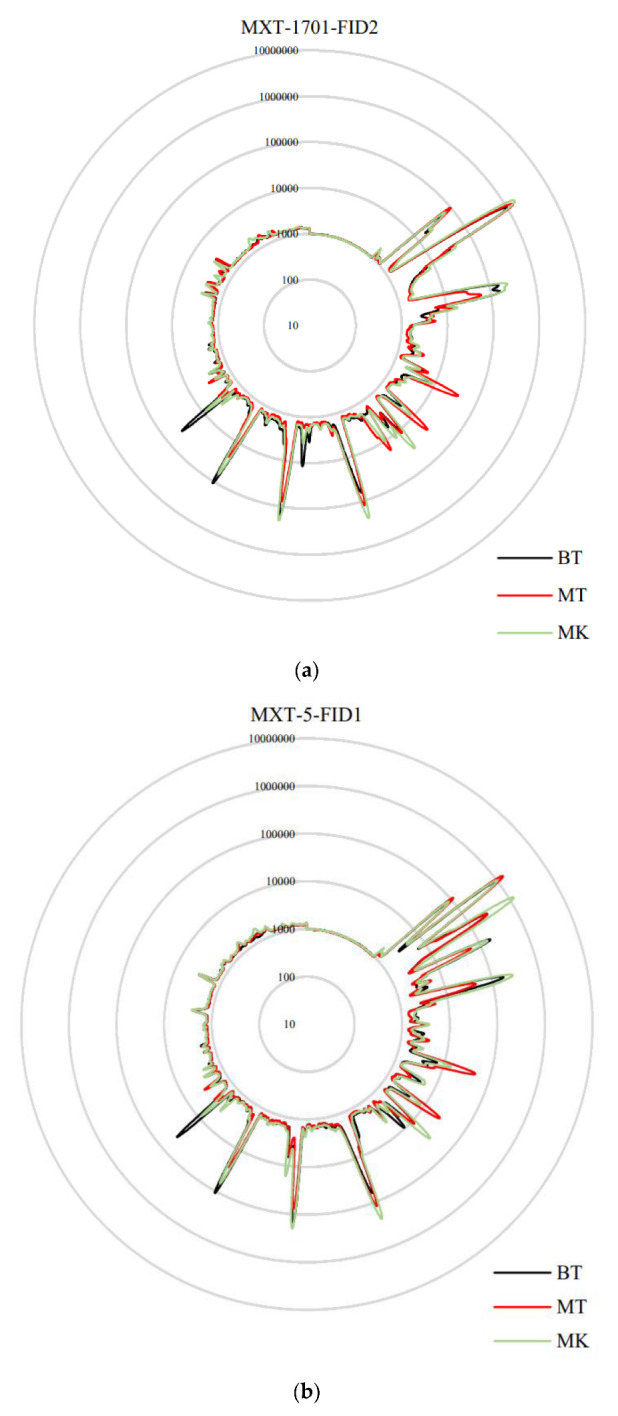
Radar map of electronic nose. (**a**) the radar map of the electronic nose under non-polar separation. (**b**) the radar map of the electronic nose under polar column separation.

**Figure 2:**
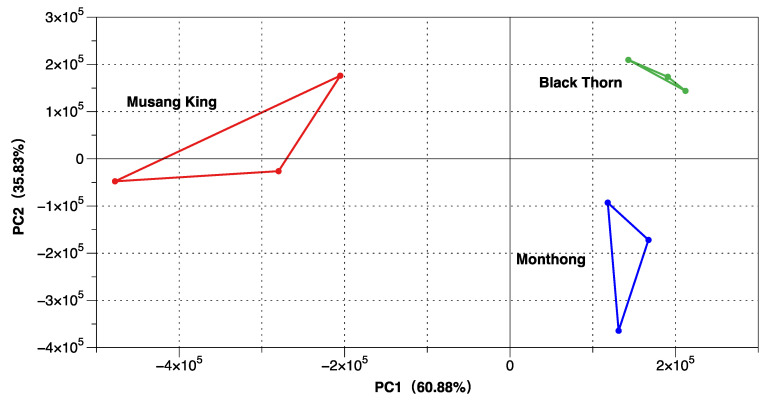
PCA of e-nose data.

The e-nose is a high-precision instrument that can detect subtle differences in the volatile components of a sample [33]. In this study, the e-nose was used to analyze the comprehensive flavor characteristic of three durians. The response intensity of the electronic nose of volatile compounds depends not only on the type of compound, but also on the concentration. Figure 1a,b shows the radar images of the electronic nose under non-polar and polar column separation, respectively. The results show that the radar fingerprint chart of the aroma of the three durians almost overlapped and that the concentrations of some volatile compounds were different. The score plot of PCA of three physicochemical indexes of different cultivars is shown in Figure 2. The results were statistically analyzed using PCA. The variance contribution rates of the first and second PCs were 60.88% and 38.83%, respectively. The total variance in contribution of the first two PCs was 99.71%. The research showed that the total variance greater than 85% indicated a rigorous classification procedure. The higher the total variance, the more the principal component can reflect the information [34]. PCA results showed that the e-nose data were stable, and the three durians could be well separated. The values of the principal Component 1 of BT and MT were similar, which indicates that they had similar flavors.

As shown in Table 3, the major taste components of the three durians were detected using the e-tongue, and the sweetness, sourness and umami tastes were detected, respectively. The e-tongue results showed that the three durians have differences regarding the perception of sweetness and umami. The greater the signal intensity value of the e-tongue, the stronger of corresponding sense of taste people feel. The critical value of sourness was −13. Below this value sourness could not be sensed. In terms of sweetness, BT had the highest sweetness and MT had the lowest sweetness, which was consistent with the LC-MS data. The total sugar concentration of BT was 257.63 μg/mg, and the total sugar concentration of MT was 138.73 μg/mg. Considering that the sweetness of different sugars is different, the reason for this result may be that the sucrose content of the Black Thorn durian is much higher than that of the other two cultivars, which masks the sweetness effect. In terms of umami, MT had the highest umami, and BT had the lowest umami. It can be seen that the taste of durian varies according to origin and cultivar.

## 3. Materials and Methods

### 3.1. Materials

BT (D200) were purchased from a local orchard in Pulau Pinang, Penang (5.354° N, 100.273° E) in August 2021. MK (D197) were purchased from a local orchard in Raub, Pahang (3.512° N, 101.405° E) in October 2021. These two cultivars naturally fell off the trees onto the ground after 120 days’ growth and the fruits were frozen in liquid nitrogen immediately. MT were purchased from a local Internet shop. MT (D159) was the most common cultivar in the Thailand market. After being picked by hand, they were immediately stored in liquid nitrogen. All these fruits were transported via SF Express to the Shanghai Institute of Technology, Fengxian District, Shanghai City, Shanghai Province, China, and kept at 4 °C on the way. The durians without any visual defects or rotting were selected. Dehusked durians were packed in aluminum foil. After being frozen in liquid nitrogen, the durian pulp was placed into a zip-lock bag and stored in a −80 °C refrigerator in order to employ it in the subsequent experiments.

### 3.2. Chemicals

Ethanol, NaOH, glucose, fructose, ribose, sucrose, maltose, methanol, chloroform, 3-nitrophenylhydrazine, formic acid, acetonitrile, propionic acid, isobutyric acid, butyric acid, oxalic acid, lactic acid, valeric acid, isovaleric acid, Malonic acid, caproic acid, isocaproic acid, fumaric acid, maleic acid, succinic acid, benzoic acid, itaconic acid, glutaric acid, malic acid, salicylic acid, caprylic acid, adipic acid, tartaric acid, pimelic acid, shikimic acid Oxalic acid, citric acid, dl-isocitric acid, quinic acid, l-Alanine, l-Arginine, l-Asparagine, l-Aspartic acid, l-Cystine, l-Glutamic acid, l-Glutamine, Glycine, l-Histidine, l-Isoleucine, l-Leucine, l-Lysine, l-Methionine, l-Phenylalanine, l-Proline, l-Serine, l-Threonine, l-Tryptophan, l-Tyrosine, l-Valine and Thiophene were purchased from Shanghai Titan technology Co., Ltd. (Shanghai, China). All reagents were analytical reagent (AR). A n-alkane (C6–C30) was purchased from Sigma-Aldrich (St. Louis, MO, USA). Pure water was obtained from a Milli-Q purification system (IQ 7000, Merck KGaA, Darmstadt, Germany).

### 3.3. Determination of Volatile Organic Compounds

SPME was used to extract the aroma components from durian pulp. The experimental methods and instrument conditions were based on the literature and some optimizations were made [26]. A total of 5 g durian pulp, 3 mL deionized water, 1 g NaCl and 1 mL 10 mg/L thiophene were added to a 15 mL headspace bottle. The fiber coating (57328-U, SUPELCO, Bellefonte, PA, USA) type was carboxen/divinybenzene/polydimethylsiloxane (CAR/DVB/PDMS). The SPME syringe was inserted into the headspace bottle in a 30 °C water bath and the fiber coating exposed for 30 min. Finally, the SPME syringe was inserted into the GC injector for desorption for 5 min at 250 °C. GC–MS (7890 GC-5973C MSD, Agilent Technologies, Santa Clara, CA, USA) was used for identification of the durian volatile compounds. HP-Innowax and DB-5 columns (60 m × 0.25 mm × 0.25 μm, Agilent Technologies, Santa Clara, CA, USA) were used for dual-column analysis. The durian volatiles were separated under the following instrumental conditions: electron ionization voltage of 70 eV, helium as carrier gas at a constant flow of 2 mL/min, inlet temperature of 250 °C, ion source temperature of 230 °C and quadrupole temperature of 150 °C. The heating program was set as follows: the initial temperature was 40 °C which was held for 6 min, the temperature was increased to 100 °C at a rate of 3 °C/min and held for 2 min, and then to 230 °C at a rate of 10 °C/min and held for 20 min. The splitless mode was chosen. The quantification of the volatile compounds was conducted by comparing the peak areas of the detected volatile organic compounds to the peak area of internal standard thiophene. Different durian samples were studied using the same analysis methods and analysis conditions. The compounds were identified by matching retention times of authentic standards, retention indices (RIs), and mass spectra in the NIST 17 database. The RIs of unknown compounds were determined by alkanes (C_6_−C_30_). The parallel experiments were performed three times.

### 3.4. Determination of Sugar Contents

HPAEC-PAD was used to determine the sugar content in durian pulp. The experimental methods and instrument conditions were based on the literature and some optimizations were made [8]. A total of 100 mg of durian pulp was accurately weighed in a 2.0 mL centrifuge tube and 700 μL 80% ethanol was added to the centrifuge tube. The sample was shaken for 2 h at 50 °C and diluted with 700 μL deionized water. Subsequently, the sample was centrifuged at 10,000 rpm for 3 min. Finally, the supernatant was transferred to a new centrifuge tube and the processed sample solution was diluted 100 times. Ion chromatography (ICS 5000, Thermo Scientific, Waltham, MA, USA) used CarboPacTM PAI (50 × 4.0 mm) liquid chromatography column. The mobile phase A was H_2_O and B was 100 mM NaOH. The injection volume was 5 μL and the flow rate was 0.5 mL/min. The column temperature was 30 °C and the elution gradient was as follows: 0 min A phase/B phase (95:5 *v*/*v*), 9 min A phase/B phase (95:5 *v*/*v*), 20 min A phase/B phase (0:100 *v*/*v*), 30 min A phase/B phase (0:100 *v*/*v*), 30.1 min A phase/B phase (95:5 *v*/*v*), 40 min A phase/B phase (95:5 *v*/*v*), 60 min A Phase/B phase (95:5 *v*/*v*). Different durian samples were studied using the same analysis methods and analysis conditions. The standard stock solution of galactose, fructose, ribose, sucrose and maltose were prepared by following concentration gradients of 0.5 μg/mL, 1 μg/mL, 5 μg/mL, 10 μg/mL, 20 μg/mL, 30 μg/mL, 40 μg/mL and 50 μg/mL. According to the calibration curve, the concentrations of glucose, fructose, ribose, sucrose and maltose in the durian sample were calculated. The software Chromeleon 7.2 (Waltham, MA, USA, 2012) was used for chromatographic data processing. Three parallel determinations were performed under the same conditions.

### 3.5. Determination of Organic Acid Contents

A Thermo Vanquish Flex UHPLC (Thermo Scientific, Sunnyvale, CA, USA) equipped with a Q-Exactive hybrid quadrupole-orbitrap mass spectrometer (Thermo Scientific, San Jose, CA, USA) was used to analyze the organic acid content in durian pulp. The experimental methods and instrument conditions were based on the literature and some optimizations were made [35]. A total of 100 mg of durian pulp was accurately weighed in a 2.0 mL centrifuge tube, and 300 μL extraction solution (methanol: chloroform = 7:3) was added to the centrifuge tube. The solution was mixed thoroughly and left to stand for about 30 min in an ice bath. A total of 200 μL of deionized water was added to the system and mixed thoroughly. The mixture was centrifuged at 12,000 rpm for 10 min at 4 °C, and the supernatant was collected. The centrifugation process was repeated once. After centrifugation, 40 μL of the mixture was mixed with 10 μL of 0.1M 3-nitrophenylhydrazine (3-NPH) at 40 °C for 30 min of incubation to complete the sample derivatization [36]. Finally, the processed sample solution was diluted 10 times. Separation of organic acid was performed on a UHPLC system equipped with a Waters BEH C18 Column (2.1 mm × 50 mm, 1.8 μm particle size). The column oven temperature was set to 40 °C. The mobile phase A was water/0.1% formic acid, and the mobile phase B was ACN/0.1% formic acid. The flow rate was 0.35 mL/min, and the injection volume was 2 μL. The elution gradient was as follows: 0.0 min A/B (90:10 *v*/*v*), 2.0 min A/B (90:10 *v*/*v*), 12.0 min A/B (10:90 *v*/*v*), 14.0 min A/B (10:90 *v*/*v*), 14.1 min A/B (90:10 *v*/*v*) and 16.0 min A/B (90:10 *v*/*v*). The UHPLC system was coupled to MS equipped with electrospray ionization (ESI) and the MS conditions were as follows: sheath gas flow rate 40 arb, auxiliary gas flow rate 10 arb, ion spray voltage −2800 V at 350 °C, ion transfer tube temperature 320 °C. The scan mode was Fullms −ms2 negative ion. Different durian samples were studied using the same analysis methods and analysis conditions. The standard stock solution of propionic acid, isobutyric acid, butyric acid, oxalic acid, (*S*)-lactic acid, valeric acid, isovaleric acid, malonic acid, hexanoic acid, 4-methylvaleric acid, fumaric acid, maleic acid, succinic acid, benzoic acid, itaconic acid, glutaric acid, d-(+)-Malic acid, salicylic acid, octanoic acid, adipic acid, d-(−)-Tartaric acid, pimelic acid, shikimic acid, citric acid, dl -isocitricacid and d-(−)-quinic acid were prepared by following concentration gradients of 1 ng/mL, 10 ng/mL, 100 ng/mL, 500 ng/mL, 1000 ng/mL, 5000 ng/mL, 10,000 ng/mL and 20,000 ng/mL, and calibration curves were constructed. According to the calibration curve, the concentration of organic acids in the durian sample were calculated. The chemical standard derivatization method was the same as the sample derivatization method. The software TraceFinder (5.1, San Jose, CA, USA, 2019) was used for data processing. Three parallel determinations were performed under the same conditions.

### 3.6. Determination of Free Amino Acids Contents

A Thermo Vanquish Flex UHPLC (Thermo Scientific, Sunnyvale, CA, USA) equipped with a Q-Exactive hybrid quadrupole-orbitrap mass spectrometer (Thermo Scientific, San Jose, CA, USA) was used to analyze the free amino acids in durian pulp. The experimental methods and instrument conditions were based on the literature and some optimizations were made [37,38,39]. The pre-column AQC (6-aminoquinolyl-N-hydroxysuccinimidyl carbamate) derivatization of amino acids was accomplished using the Waters AccQ·FluorTM (Waters Corporation, Milford, MA, USA) kit. The derivatization of AccQ·Fluor reagent, the derivatization of amino acid standard samples, the construction of the external standard calibration curve and the derivatization of the durian sample (50 mg) were strictly performed in accordance with instruction manual from Waters (Waters AccQ Tag Chemistry Package Instruction Manual, Revision 1). The processed sample solution was diluted nine times. Separation of amino acids was performed on a UHPLC system equipped with a Waters BEH C18 Column (2.1 mm × 50 mm, 1.7 μm particle size). The column oven temperature was set to 55 °C. The mobile phase A was water/0.1% formic acid, and the mobile phase B was ACN/0.1% formic acid. The flow rate was 0.35 mL/min, and the injection volume was 1 μL. The elution gradient was as follows: 0.0 min A/B (95:5 *v*/*v*), 5.5 min A/B (90:10 *v*/*v*), 7.7 min A/B (75:25 *v*/*v*), 8.0 min A/B (40:60 *v*/*v*), 8.5 min A/B (95:5 *v*/*v*) and 13.0 min A/B (95:5 *v*/*v*). The UHPLC system was coupled to MS equipped with electrospray ionization (ESI) and the MS conditions were as follows: sheath gas flow rate 40 arb, auxiliary gas flow rate 10 arb, ion spray voltage + 3000 V at 350 °C, ion transfer tube temperature 320 °C. The scan mode was single ion detection (SIM). Different durian samples were studied using the same analysis methods and analysis conditions. The software TraceFinder (5.1, San Jose, CA, USA, 2019) was used for data processing. Three parallel determinations were performed under the same conditions.

### 3.7. E-Tongue Analysis

The e-tongue (TS-5000Z, Insent Company, Fukuoka ken, Japan) was used to analyze the taste components of the three durians. Sensor CA0, AAE and Gl1 were used to detect sourness, umami and sweetness taste. The experimental instrument settings and experimental methods referred to the previous literature [40]. A total of 5 g durian pulp and 40 mL deionized water were added to a 50 mL centrifuge tube. The mixture was filtered with refrigerated centrifuge (4 °C, 8000 rpm, 10 min), and 30 mL of the supernatant was taken for detecting. The parallel experiments were performed four times.

### 3.8. Electronic Nose Analysis

The electronic nose (Alpha MOS, Toulouse, France) was used to quickly identify the aroma of durian. The parameter settings of the instrument refer to the literature [41]. The method of extracting durian pulp aroma was the same as the method for SPME above. The parallel experiments were performed three times.

## 4. Conclusions

This study systematically investigated the volatile compounds and taste components of durians (BT, MT and MK). The types of organic acids and free amino acids in the three durians are almost the same, but the content of each substance is not the same. The number of volatile compounds in BT is less than that in the other two cultivars, but the total sugar content is higher than in the other two cultivars. The results of e-nose, e-tongue and PCA can effectively distinguish different durians. The evaluation results are in good agreement with the LC-MS data. Compared with an artificial sensory system, they are quick and objective evaluation methods. These results provide evidence for the flavor formation mechanisms of different durians, which need to be further studied. The volatile aroma compounds, organic acids, sugars and amino acids of durian have a significant impact on the quality and popularity of durian.

## Figures and Tables

**Table 1:** Qualitative and quantitative analysis of volatile compounds in durian pulp.

No.	Compound	CAS	RI		Identification ^c^	Concentration (μg/kg)					
			HP-Innowax ^a^	DB-5 ^b^		Black Thorn	RSD ^d^ (%)	Monthong	RSD (%)	Musang King	RSD (%)
1	ethyl mercaptan	75-08-1	756	<600	St RI MS	364.17	12.80	1617.98	13.63	3125.34	7.76
2	propyl mercaptan	107-03-9	835	<600	St RI MS	174.28	13.99	-	-	218.54	9.92
3	ethyl methyl sulfide	624-89-5	856	612	St RI MS	-	-	-	-	2389.15	9.11
4	ethyl acetate	141-78-6	873	<600	St RI MS	-	-	1722.75	1.53	-	-
5	ethyl propionate	105-37-3	950	681	St RI MS	61.43	12.48	-	-	-	-
6	ethyl isobutyrate	97-62-1	962	740	St RI MS	-	-	1014.01	3.51	341.15	6.90
7	diacetyl	431-03-8	988	<600	St RI MS	-	-	2075.81	2.05	1537.33	9.54
8	2-pentanone	107-87-9	988	662	St RI MS	-	-	74.65	10.80	-	-
9	methyl 2-methyl butyrate	868-57-5	1009	762	St RI MS	146.05	4.21	468.65	2.76	964.96	1.81
10	ethyl butyrate	105-54-4	1021	785	St RI MS	-	-	559.80	13.00	-	-
11	propyl alcohol	71-23-8	1038	<600	St RI MS	1012.22	6.32	2456.31	13.88	3739.34	5.41
12	ethyl 2-methyl butyrate	7452-79-1	1053	834	St RI MS	1722.95	8.10	11,680.31	5.26	14,484.20	11.36
13	dipropyl sulfide	111-47-7	1076	879	St RI MS	-	-	-	-	20.85	6.60
14	S-ethyl thioacetate	625-60-5	1095	755	St RI MS	-	-	-	-	74.39	10.03
15	diethyl carbonate	105-58-8	1105	766	St RI MS	-	-	564.51	2.67	-	-
16	methyl (*E*)-2-butenoate	623-43-8	1109	746	St RI MS	-	-	83.90	5.95	37.58	4.26
17	ethyl valerate	539-82-2	1126	880	St RI MS	-	-	163.79	2.88	-	-
18	propyl 2-methyl butyrate	37064-20-3	1131	931	St RI MS	1818.42	11.66	1137.25	8.18	2880.98	7.66
19	ethyl methyl disulfide	20333-39-5	1148	816	St RI MS	124.34	6.91	526.51	8.96	1191.53	6.72
20	ethyl (*Z*)-crotonate	6776-19-8	1150	754	St RI MS	108.13	6.61	-	-	-	-
21	propyl isovalerate	557-00-6	1152	933	St RI MS	43.44	13.13	146.97	4.65	-	-
22	butyl alcohol	71-36-3	1154	653	St RI MS	-	-	631.74	13.29	305.87	1.89
23	heptanal	111-71-7	1171	882	St RI MS	-	-	71.22	13.91	-	-
24	(*E*)-methyl tiglate	6622-76-0	1180	858	St RI MS	37.68	11.62	49.20	10.28	-	-
25	dextro-limonene	5989-27-5	1196	1015	St RI MS	-	-	72.90	4.52	40.06	13.11
26	2-methyl-1-butanol	137-32-6	1201	725	St RI MS	290.26	12.07	990.66	3.33	-	-
27	isoamyl alcohol	123-51-3	1203	721	St RI MS	-	-	550.31	5.71	1637.92	5.67
28	diethyl disulfide	110-81-6	1212	907	St RI MS	17,777.04	3.00	8665.01	10.52	27,253.26	11.76
29	methyl propyl disulfide	2179-60-4	1221	917	St RI MS	155.98	9.20	102.43	7.21	159.36	5.87
30	ethyl hexanoate	123-66-0	1226	979	St RI MS	355.56	10.66	1527.06	4.70	352.27	5.51
31	(*E*)-ethyl tiglate	5837-78-5	1228	925	St RI MS	430.37	7.21	-	-	204.04	9.78
32	amyl alcohol	71-41-0	1239	750	St RI MS	-	-	63.27	6.20	79.48	10.65
33	1-octanal	124-13-0	1281	984	St RI MS	-	-	81.84	8.97	-	-
34	acetoin	513-86-0	1283	691	St RI MS	-	-	1240.91	11.11	655.79	3.72
35	propyl tiglate	61692-83-9	1331	1028	St RI MS	262.85	2.32	74.17	2.99	28.18	5.02
36	hexyl formate	629-33-4	1350	901	St RI MS	-	-	264.43	8.94	-	-
37	hexanol	111-27-3	1363	851	St RI MS	106.54	3.80	-	-	505.16	2.31
38	dipropyl disulfide	629-19-6	1367	1101	St RI MS	538.15	11.86	61.25	4.07	511.46	2.68
39	methyl octanoate	111-11-5	1381	1113	St RI MS	53.80	8.18	110.43	6.70	43.22	9.04
40	nonanal	124-19-6	1392	1088	St RI MS	-	-	106.20	8.03	121.50	10.17
41	hexyl 2-methyl butyrate	10032-15-2	1431	1227	St RI MS	29.06	13.46	-	-	-	-
42	ethyl octanoate	106-32-1	1441	1178	St RI MS	265.37	2.84	860.81	3.19	173.15	6.34
43	diethyl trisulfide	3600-24-6	1520	1134	St RI MS	2514.78	3.46	1013.71	13.49	3203.47	11.51
44	3-hydroxybutyric acid ethyl ester	5405-41-4	1524	923	St RI MS	-	-	409.98	8.75	64.70	7.23
45	3,5-dimethyl-1,2,4-trithiolane	23654-92-4	1599	1126	St RI MS	336.17	11.02	167.21	1.33	264.36	3.39
46	beta-caryophyllene	87-44-5	1605	1408	St RI MS	-	-	-	-	20.87	1.37
47	ethyl decanoate	110-38-3	1649	1380	St RI MS	-	-	57.82	7.87	19.08	11.35
48	dipropyl trisulfide	6028-61-1	1712	1319	St RI MS	49.08	8.33	-	-	37.57	9.64
49	acetaldehyde diethyl mercaptal	14252-42-7	2223	1317	St RI MS	343.83	12.56	20.55	10.32	889.76	6.23

^a^ Retention index of compounds on the HP-Innowax. ^b^ Retention index of compounds on the DB-5. ^c^ RI: retention index; Std: authentic standards; MS: mass spectrometry. ^d^ RSD: relative standard deviation.

**Table 2:** Sugar, organic acid, and amino acid contents of three durian cultivars.

Attributes	Durian Cultivars					
	Black Thorn	RSD ^a^ (%)	Musang King	RSD (%)	Monthong	RSD (%)
Sugar (μg/mg)						
Glucose	10.49	1.1	34.26	1.90	30.91	1.46
Fructose	10.05	0.5	49.45	1.59	43.70	1.27
Ribose	0.69	5.03	-	-	-	-
Sucrose	235.34	0.89	111.53	1.17	57.26	0.96
Maltose	1.06	1.03	20.76	1.28	6.87	0.34
Organic acid (ng/mg)						
Propionic acid	2210.56	4.99	1109.35	4.13	275.93	1.84
Isobutyric acid	49.83	1.35	8.18	1.73	29.48	1.62
Butyric acid	117.97	2.51	77.01	3.13	49.84	2.91
Oxalic acid	798.03	2.42	263.26	1.59	389.03	1.49
lactic acid	34.22	4.46	41.64	0.64	369.25	2.97
Valeric acid	5.17	4.28	5.36	0.19	3.23	2.71
Isovaleric acid	87.38	4.08	26.75	0.69	30.24	4.22
Malonic acid	14.45	2.69	29.83	1.95	8.40	0.45
Hexanoic Acid	55.52	3.00	45.53	0.81	38.58	0.03
4-Methylvaleric Acid	61.59	0.17	51.66	4.16	43.26	2.03
Fumaric acid	260.76	2.06	444.80	1.17	281.58	1.40
Maleic acid	1316.30	3.83	2245.44	0.13	1420.47	4.87
Succinic acid	11,453.95	3.10	8442.83	2.79	4681.37	0.26
Benzoic acid	4.23	0.76	10.10	3.41	6.75	0.86
Itaconic acid	1.06	3.37	0.15	4.09	0.25	0.17
Glutaric Acid	94.26	4.58	103.23	4.61	58.34	2.30
Malic acid	3888.95	0.48	5986.80	2.05	4180.52	3.70
Salicylic acid	1.04	4.43	0.82	4.81	0.67	2.53
Octanoic Acid	27.02	2.87	30.16	1.14	35.95	0.79
Adipic acid	42.66	0.87	202.74	0.98	24.35	0.32
Tartaric acid	72.44	1.57	125.45	1.19	221.67	4.27
Pimelic Acid	39.89	2.96	40.51	3.43	44.09	0.62
Shikimic Acid	28.11	0.57	29.00	2.09	64.06	2.14
Citric acid	4875.13	2.58	1141.72	1.52	1897.57	0.55
Isocitricacid	212.97	0.12	47.38	5.62	72.81	3.86
Quinic acid	99.10	0.32	4.08	2.85	43.12	1.91
Acetic acid	2934.75	1.22	5490.22	4.12	3521.15	2.18
Amino acids (ng/mg)						
l-Alanine	6451.55	2.45	3897.17	1.43	6019.09	3.94
l-Arginine	1526.99	1.55	1267.24	2.23	1108.34	1.63
l-Asparagine	65.71	3.62	39.83	1.97	42.86	3.73
l-Aspartic acid	6708.92	5.74	4634.31	2.47	3884.03	1.36
l-Cystine	87.47	3.61	104.66	2.29	63.88	0.66
l-Glutamic acid	3849.50	2.24	3462.46	0.34	3040.48	0.93
l-Glutamine	0.99	2.71	0.75	3.68	1.91	3.59
Glycine	1796.68	0.60	1617.87	0.44	1506.77	4.59
l-Histidine	615.19	4.47	520.96	1.53	460.74	4.86
l-Isoleucine	1186.12	1.83	1189.70	3.01	1109.90	1.83
l-Leucine	2156.67	4.03	2137.24	1.64	1903.56	2.98
l-Lysine	1614.67	0.26	1446.72	0.92	1381.04	2.77
l-Methionine	54.18	3.49	63.62	2.30	38.23	3.03
l-Phenylalanine	1454.20	4.51	1243.40	1.34	1093.01	1.76
l-Proline	1699.47	0.72	1499.98	3.21	1440.28	0.12
l-Serine	1580.29	1.10	1390.38	1.87	1305.95	2.57
l-Threonine	1177.47	2.20	1130.76	1.71	1016.17	4.58
l-Tryptophan	-	-	0.15	4.26	-	-
l-Tyrosine	875.93	3.41	721.99	2.11	654.02	4.29
l-Valine	1524.03	3.46	1454.92	0.31	1354.35	2.19

^a^ RSD, relative standard deviation.

**Table 3:** Electronic tongue results of samples.

	Black Thorn	RSD ^a^ (%)	Musang King	RSD (%)	Monthong	RSD (%)
Sweetness	22.33	0.48	22.03	0.51	21.02	0.59
Sourness	−25.04	2.13	−26.26	2.52	−26.39	2.02
Umami	10.88	1.02	11.34	0.81	11.52	0.69

^a^ RSD, relative standard deviation.

## Data Availability

The data presented in this study are available on request from the corresponding author. The data are not publicly available due to sensitivity.

## Sample Availability

Samples of the compounds are available from the authors.
